# Supporting clinical rules engine in the adjustment of medication (SCREAM): protocol of a multicentre, prospective, randomised study

**DOI:** 10.1186/s12877-017-0426-3

**Published:** 2017-01-26

**Authors:** Carlota Mestres Gonzalvo, Hugo A. J. M. de Wit, Brigit P. C. van Oijen, Kim P. G. M. Hurkens, Rob Janknegt, Jos M. G. A. Schols, Wubbo J. Mulder, Frans R. Verhey, Bjorn Winkens, Paul-Hugo M. van der Kuy

**Affiliations:** 1 Department of Clinical Pharmacy and Toxicology, Zuyderland Medical Centre, Sittard, The Netherlands; 2 Department of Clinical Pharmacy and Toxicology, Zuyderland Medical Centre, Heerlen, The Netherlands; 3 Department of Internal Medicine, Zuyderland Medical Centre, Heerlen, The Netherlands; 4 Department of Family Medicine and Department of Health Services Research, School for Public Health and Primary Care (CAPHRI), Maastricht University, Maastricht, The Netherlands; 5 Department of Internal Medicine, Maastricht University Medical Centre, Maastricht, The Netherlands; 6 Department of Psychiatry and Neuropsychology, Alzheimer Centre Limburg/School for Mental Health and Neurosciences (MHeNS), Maastricht University, Maastricht, The Netherlands; 7 Department of Methodology and Statistics, School for Public Health and Primary Care (CAPHRI), Maastricht University, Maastricht, The Netherlands

**Keywords:** Polypharmacy, Medication therapy management, Decision support systems management, Aged, Medication review

## Abstract

**Background:**

In the nursing home population, it is estimated that 1 in every 3 patients is polymedicated and given their considerable frailty, these patients are especially prone to adverse drug reactions. Clinical pharmacist-led medication reviews are considered successful interventions to improve medication safety in the inpatient setting. Due to the limited available evidence concerning the benefits of medication reviews performed in the nursing home setting, we propose a study aiming to demonstrate a positive effect that a clinical decision support system, as a health care intervention, may have on the target population. The primary objective of this study is to reduce the number of patients with at least one event when using the clinical decision support system compared to the regular care. These events consist of hospital referrals, delirium, falls, and/or deaths.

**Method/Design:**

This study is a multicentre, prospective, randomised study with a cluster group design. The randomisation will be per main nursing home physician and stratified per ward (somatic and psychogeriatric). In the intervention group the clinical decision support system will be used to screen medication list, laboratory values and medical history in order to obtain potential clinical relevant remarks. The remarks will be sent to the main physician and feedback will be provided whether the advice was followed or not. In the control group regular care will be applied.

**Discussion:**

We strongly believe that by using a clinical decision support system, medication reviews are performed in a standardised way which leads to comparable results between patients. In addition, using a clinical decision support system eliminates the time factor to perform medication reviews as the major problems related to medication, laboratory values, indications and/or established patient characteristics will be directly available. In this way, and in order to make the medication review process complete, consultation within healthcare professionals and/or the patient itself will be time effective and the medication surveillance could be performed around the clock.

**Trial registration:**

The Netherlands National Trial Register NTR5165. Registered 2nd April 2015.

## Background

Polypharmacy is defined as the use of more than a certain number of drugs irrespective of their appropriateness [1, 2]. In the Netherlands, it has been defined as the chronic use of 5 or more drugs from different therapeutic groups or subgroups [3]. In the nursing home population, it is estimated that 1 in every 3 patients is polymedicated [4] and given their considerable frailty, these patients are extra prone to adverse drug reactions. In addition, their management is often challenging given the comorbidities and/or complex organ function impairment [1, 2, 5–8]. Furthermore, polymedicated patients are also at risk of suffering from inappropriate prescribing in the form of underprescription. It has been demonstrated that underprescription increases significantly with the number of medicines used [9]. This situation strengthens the need for routine medication reviews and treatment optimisation [10, 11].

Clinical pharmacist-led medication reviews are considered successful interventions to improve medication safety in the inpatient setting. However, there is limited available evidence of the effects concerning comparable interventions performed in the outpatient setting [1, 12, 13]. In addition, few studies have evaluated health related outcomes resulting from clinical pharmacist interventions in nursing homes. Nevertheless, it has been suggested that most of these studies had major limitations: no control group, no clinical outcome measures, inadequate use of nursing staff to influence change, and data analysis by drug use per provider rather than drug use per patient [6, 8, 10, 11, 13, 14]. Some of these studies were randomised controlled trials performed in the nursing home setting by means of a clinical pharmacists-led medication review; some of them measured the effect of multidisciplinary case conference [6, 15]. In other studies, pharmacists performed the medication reviews and sent suggestions to physicians [8, 10, 16]. Nevertheless, some improvements in patient outcomes have been described [8, 10]. The results from these studies are difficult to compare due to the large differences with respect to the interventions applied, the outcomes studied, the settings, and duration of follow-up after the medication review.

Pharmacotherapy optimisation in nursing home patients relies on the development and assessment of novel healthcare interventions [11]. It is suggested that performing a standardised intervention could potentially lead to a successful medication review; this intervention necessitates pharmacists and physicians collaboration, it should include the complete medical and drug history, and fully availability of laboratory values should be guaranteed [10, 12, 13, 17–20].

In the Netherlands, the Dutch Healthcare Inspectorate (IGZ: Inspectie voor de Gezondheidszorg) expects that a medication review is performed by a physician and a pharmacist in all residents of nursing homes yearly; however, this advice implies substantial extra workload for the involved health care professionals. In addition, in this medication review the information given by the nursing staff and the patient him/herself should also be taken into account.

From our experience, medication reviews involve a time consuming process that takes an average of 90 min per patient. When considering a nursing home of about 150 patients, 450 h a year would have to be dedicated at performing medications reviews.

In daily practice, this unfortunate situation leads to a non-continuous medication review process implying major consequences that may range from an increased number of potential adverse drug reactions, unnecessary hospitalisations and, at worst, death.

Computerised clinical decision support systems (CCDSS) can be defined as decision-aiding tools which provide health care professionals with clinical knowledge and patient-related information, intelligently filtered or presented at appropriate times, so as to enhance patient care [21–23]. Within the SCREEN project (Supporting Clinical Rules in the Evaluation of Elderly patients with Neuropsychiatric disorders), a CCDSS named Clinical Rule Reporter (CRR) has been developed. This system currently analyzes, independently of the applied prescribing software, the medication used by patients in relation to their co-medication, the laboratory data (including renal function), and other relevant clinical data like diagnosis and comorbidities [24]. The CRR combines the clinical rules (algorithms) with the medication list, patient characteristics and laboratory values of the patients in order to obtain concrete advices. These clinical rules or algorithms work with triggers that identify drug related problems like renal or liver dysfunction as well as the need of new medication (stomach protection or laxative agents), the necessity to stop a certain drug or decrease the dose according to age, etc.

Due to the lack of evidence concerning the benefits of medication reviews performed in the nursing home setting, we propose a study aiming to demonstrate a positive effect that the CRR, as a health care intervention, may have on the target population. This population consists of older people (≥65 years) with a high risk of suffering harm when using inappropriate drugs. By this we mean people living in nursing home facilities; these people often suffer from polymedication among other risk factors such as multimorbidity, impaired cognition, renal dysfunction, and increased risk of falling.

The primary objective of this study is to reduce the number of patients with at least one event when using the CRR compared to the regular care. These events consist of hospital referrals, delirium, falls, and/or deaths. Secondary objectives will also be evaluated, including: the analysis within a centre to account for possible differences concerning regular care, the separate analysis for psychogeriatric and somatic wards, the analysis for medication related events (hospital referrals, delirium, falls, and/or deaths), the separate analysis for each of the parameters included in the combined endpoint, the analysis of the quality of life EQ-5D, the analysis of the MAI (Medication Appropriate Index), and finally, a cost analysis.

## Methods/Design

### Study design

The Supporting Clinical Rules Engine in the Adjustment of Medication (SCREAM) study is a multicentre, prospective, randomised study with a cluster group design. The randomisation will be per main nursing home physician and stratified per ward (somatic and psychogeriatric). This study will be blinded for physicians and for patients; physicians will be emphatically requested not to discuss with each other about the study to avoid bias. The study follows the CONSORT guidelines.

### Overall study design

In order to use the CRR, the nursing homes will have to provide the medication list, the patient characteristics and the laboratory values for each patient in a digital format.

Taking into account the extra workload for the investigators, there will be a predefined day for each nursing home to send the files: nursing home A sends the files on Mondays, nursing home B sends the files on Tuesday, and so on.

All nursing homes will send the patient data both for control and intervention groups.

The randomisation will be performed by two of the authors (BvO, CMG). The randomisation will be per main nursing home physician and stratified per ward. Physician A will be randomised in the control group and physician B on the intervention group, taking into account that the amount of patients in each group should be approximately the same.

#### Intervention group

The datasets will be screened through the CRR on a weekly basis. The messages delivered by the CRR will be sent via mail to the specific physicians. Each remark will be sent on a separate mail in a standardised way. In response to the report, the physician will send a feedback message within 36 h indicating, in a standardised way, whether:

- the advice was not followed
- the advice was followed
- the advice was changed.

After receiving this feedback, the investigators will process it in the CRR, in order to create the database for the study.

Additionally, regular care will be also applied. That is according to the Dutch Healthcare Inspectorate, a yearly medication review with a physician and a pharmacist, even though there is a substantial variation [25], For the centres included in this study there are no dedicated clinical pharmacist working in the nursing home.

#### Control group

In the control group patients will receive regular care (yearly medication review). In addition, these patients will also be screened using the CRR to obtain data that could serve for future evaluations within the project (for instance to compare how many advices would have been sent from the control group, the difference in remarks, etc.). However, this screening will be performed via a filter and the investigators will neither see nor evaluate any remark. These alerts will only be unblinded at the end of the study.

**Fig. 1:**
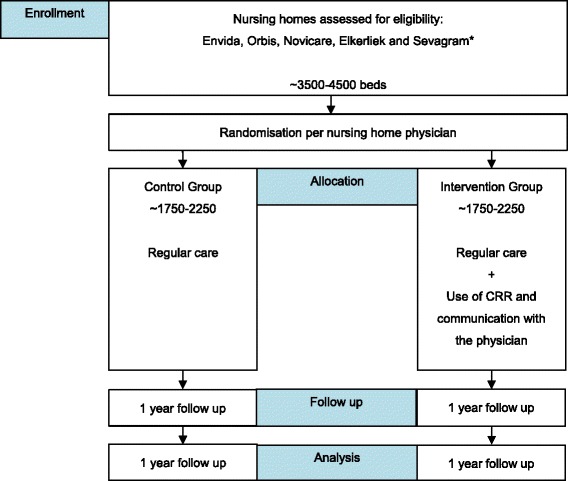
Schematic study design. *Other possible centres Amsterdam and Nijmegen

**Fig. 2:**
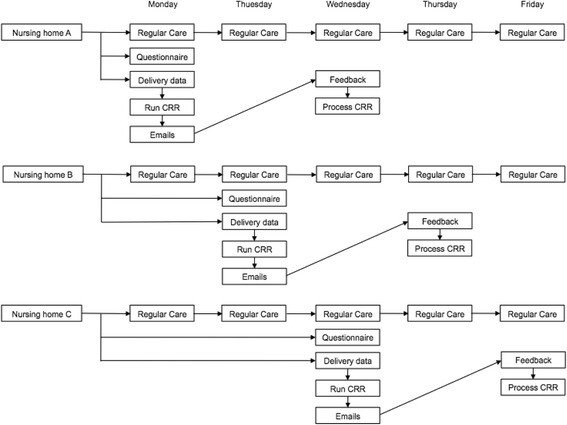
Study schedule

In addition, for both control and intervention group, the physicians will report any events including: hospital admission, specialist visit, emergency department visit, falls, delirium and death, via a questionnaire. This questionnaire will also include questions to know whether a medication review has been performed and how much time this medication review cost. Physicians will also report if there is any new patient. These electronic questionnaire will be sent by Google Drive via email weekly. At the end of the study, the physicians in the intervention group will receive a mail asking how much time, in average, they need to answer the remarks which are sent from the CRR. Figures 1 and 2.

### Endpoints

#### Primary endpoint

The primary outcome variable in this study is the proportion of patients with at least one of the events, including hospital referrals (i.e. referral to a specialist, emergency department visit and hospital admission), delirium, falls, and/or deaths. All these events will be reported by the nursing home physician via the electronic questionnaire. To this end the study will assess the differences between regular care (control group) and regular care + CRR (intervention group).

#### Secondary endpoints

As secondary endpoints, the same outcome variable will be used to analyse the possible differences between institutions, to separately analyse psychogeriatric and somatic wards, to analyse the medication related events, and to separately analyse each of the parameters included in the combined endpoint (hospital referrals, delirium, falls, and/or deaths).

In order to get this information, physicians will be asked to report any events including: hospital admission, polyclinic visit, emergency department visit, falls, delirium and death. These questions will be asked via an electronic questionnaire via Google Drive. The assessment of whether the event is or could be drug related or not will be done exclusively by the physician.

The quality of life will be measured using the EQ-5D questionnaire both for patients in the control group and patients in the intervention group. The questionnaire will be performed at the end of the study (i.e. after one year follow-up), both for psychogeriatric and somatic patients. The results will be compared between intervention and control group. For both patients groups a caregiver/nurse will answer the questionnaire.

In addition the analysis of the MAI and the cost evaluation will also be performed for both control and intervention group.

### Setting

Nursing homes in the Netherlands will be invited to participate in the study; these nursing homes should be able to deliver the medication data and the laboratory data electronically. In case the data would come from the hospital in the neighbourhood, this hospital would have to agree on providing the data. If a nursing home meets these requirements, it is eligible for participation in the present study.

### Population

Nursing home residents; the total study population is estimated to have a total of 3500–4500 patients. This wide range in amount of patients comes from the fact that patients will not be included singly but as complete nursing homes. In addition, enough patients should be included to ensure reliable results taking into account possible loss to follow up.

### Inclusion criteria

Residents living in a nursing home in the Netherlands.

The nursing homes are able to deliver the medication and lab data electronically.

### Participating centres

Zuyderland Medical Centre in Sittard-Geleen (coordinating centre), Envida in Maastricht, Sevagram in Heerlen, Elkerliek in Helmond, and Novicare in different locations. Other centres will be invited and included when the requirements are fulfilled (Amsterdam and Nijmegen).

### Randomisation, blinding and treatment allocation

#### Randomisation

per main nursing home physician. The randomisation will be stratified per ward (somatic and psychogeriatric). In case the physician would be absent the suitable option will be followed:

Absence ≤ 6 weeks intervention group: the mails with the messages obtained from the CRR will still be sent to the main physician. If the replacing physician would also participate in the study, he will not get any mails for the group of patients for which he/she is the replacing physician during this period. It is assumed that if the replacing physician is included in the intervention group, he could apply the mails from his own group to all patients. If the replacing physician is included in the control group, it is assumed that no interventions will be performed.

Absence > 6 weeks intervention group: the mails will be sent to the replacing physician; if the replacing physician would be one of the physicians already included and randomised in the control group, the replacing physician will get the mails only for the patients in the intervention group.

#### Blinding

Blinded for patients; In addition, physicians will be emphatically requested not to talk about the emails.

#### Treatment allocation

If a patient dies or moves to another institution, the replacing patient will not take over the place in the study. Death is one of the endpoints for the study and so the study would be completed for that patient; moving to another institution will be considered as loss to follow-up. To account for these patients the physicians will have to report every time a new patient gets in the nursing home, in this way a filter can be applied to not analyse these new patients.

### Time schedule

Recruitment started in June 2013; the target population, 3500–4500 patients, is expected to be accomplished in June 2016. The different centres can start with the study at different times. Each centre will be followed for a period of 1 year and afterwards the data analysis will start.

### Organisation

Each participating centre has provided a contact person who will be in charge of coordinating the study in their centres. The investigators have regular contact with these coordinating people to confirm the fulfilment of the inclusion criteria, the adherence to the study protocol, and to provide support or additional information when necessary.

### Cost analysis

A cost analysis will be performed for both groups (control and intervention).

#### Hospital costs

The analysis will take into account the number of hospitalisations or hospital referrals, consisting of personnel (physician, nurse, pharmacists, etc.), material and equipment costs. These costs will be based on study patients records and standard rates.

#### Costs outside the hospital

This analysis will also take into account the healthcare costs outside the hospital like the addition of new medication.

### Sample size calculation

Calculation of the total number (one event per patient). The aim is to reduce the number of patients with at least one event with 25% by using the CRR compared to the regular care. These events consist of medication related hospital referrals, delirium, falls, and/or deaths.

In order to calculate the sample size a pilot study was performed. Nursing homes physicians from the region (Envida and Zuyderland) have informed, via an electronic questionnaire, about any hospital referrals, delirium and/or falls within their patients. In addition, they stated whether these events could be medication related. This pilot study has lasted for 5 months. No patient information was given.

The pilot study showed a proportion of patients with at least one event (combination of fall, delirium, hospital referral, and death) in the control group of 0.16 and a mean number of patients per physician of 56.

Assuming a proportion of patients with at least 1 event during 1 year follow-up of 0.20 in the control group, a 25% reduction by using the CRR compared to regular care, i.e. proportion reduces from 0.20 to 0.15, and a two-sided significance level (α) of 0.05, the number of patients per group required to detect an effect with 80% power equals 906. Accounting for the design effect (randomisation per physician; DE = 1 + (m-1)*ICC), where we assume an intra-class correlation coefficient (ICC) of 0.01, a mean number of patients per physician (m) of 56 (pilot study), and a 10% dropout rate, the required number of patients increases to 1562 per group.

We assumed a higher proportion of patients with at least one event in the control group (0.20) than the one found in the pilot study (0.16), because the number of falls were underreported in the pilot study and the follow-up duration is now longer, i.e. one year instead of five months.

### Statistical analysis

To account for the cluster randomisation (physicians are randomised, where patients are clustered within physicians), all linear and logistic mixed effects analyses are performed with physicians as random factor.

#### Primary study parameters

To detect a difference in proportions of the primary outcome (composite endpoint consisting of hospital referrals, delirium, falls, and/or deaths) between the groups (control versus intervention), logistic mixed effects analysis are applied with the following fixed factors: group (control or intervention), nursing home organisation (Envida, Zuyderland, Sevagram or Novicare), type of ward (psychogeriatric or somatic) and other variables related to the outcome, like age and sex.

#### Secondary study parameters

For the subgroup analyses (within nursing home organisation or within type of ward), the same analysis method is applied as for the primary outcome variable, excluding the variable that indicates the subgroups.

For the other endpoints, linear or logistic mixed models are used, depending on the type of outcome (numerical or binary, respectively). Furthermore, the same fixed effects as for the primary outcome are included.

## Discussion

Other studies have mainly focused on surrogate outcomes as primary endpoint. These endpoints, such as reduction of drugs, MAI or drug costs, fail at showing clinical outcomes [10, 12, 15, 26–29]. In the present study, we are focusing both on hard endpoints (i.e. patient relevant outcomes), and surrogate outcomes. The primary endpoint, however, is a combined set of hard endpoints with a clear clinical outcome. For this reason, the duration of this study is one year; other studies not using hard endpoints have shorter study periods [6, 8, 10, 29]. Furthermore, this study is a multicentre study including over 3000 patients making it a relatively large study in comparison with other studies [6, 8, 10, 27, 28].

A major discussion point with other articles is the fact that a great number of studies focus on reducing the amount of prescribed drugs whereas the focus should be on optimising the prescribed drugs (rationalistic pharmacotherapy). This fact enlightens the paradoxically relation between polypharmacy and underprescribing as it might be confronting to add new medication to an already polymedicated patient whereas reducing medication might seem the most logical way to perform [9]. For some patients, optimising the medication will imply reducing the number of drugs, for other patients it will be the changing of some drugs or adding some drugs [30].

We strongly believe that by using a CCDSS, medication reviews are performed in a standardised way which leads to comparable results between patients. In addition, using a CCDSS eliminates the time factor to perform medication reviews as the major problems related to medication, laboratory values, indications and/or established patient characteristics will be directly available. In this way, and in order to make the medication review process complete, consultation within healthcare professionals and/or the patient itself will be time effective and the medication surveillance could be performed around the clock. Especially for polymedicated patients, like nursing home patients, this system provides a hand full of advantages to provide continuous surveillance, improving in this way patient care.

## Abbreviations

CCDSS: (Computerised clinical decision support system)
CRR: (Clinical rule reporter)
ICC: (Intra-class correlation coefficient)
IGZ: (Inspectie voor de gezondheidszorg/dutch health inspectorate)
MAI: (Medication appropriate index)
METC: (Medisch Ethische toetsingscommissie/medical ethical committee)
SCREAM: (Supporting clinical rules engine in the adjustment of medication)
SCREEN: (Supporting clinical rules in the evaluation of elderly patients with neuropsychiatric disorders)
